# Avances en el control del tabaco en la Región de las Américas 2020

**DOI:** 10.26633/RPSP.2021.94

**Published:** 2021-08-12

**Authors:** Rosa Carolina Sandoval, Adriana Bacelar Gomes, Maxime Roche, Natalia Parra, Francisco Armada

**Affiliations:** 1 Organización Panamericana de la Salud Washington, DC Estados Unidos de América Organización Panamericana de la Salud, Washington, DC, Estados Unidos de América; 2 Campaign for Tobacco-Free Kids Washington, DC Estados Unidos de América Campaign for Tobacco-Free Kids, Washington, DC, Estados Unidos de América.

**Keywords:** Legislación como asunto, tabaco, enfermedades no transmisibles, políticas públicas de salud, factores de riesgo, Américas, Legislation as topic, tobacco, noncommunicable diseases, public health policy, risk factors, Americas, Legislação como assunto, tabaco, doenças não transmissíveis, políticas públicas de saúde, fatores de risco, América

## Abstract

Se describe el estado actual y los avances en la aplicación de las medidas de control del tabaco contenidas en la Estrategia y Plan de Acción para Fortalecer el Control del Tabaco en la Región de las Américas 2018-2022 y se identifican los logros alcanzados entre los años 2016 y 2020 y los retos que aún se deben enfrentar para cumplir las metas previstas. Para ello se utilizaron los datos del Informe de la Organización Mundial de la Salud (OMS) sobre la Epidemia Mundial de Tabaquismo de los años 2015, 2017 y 2019, así como las normativas nacionales para determinar su consistencia con los criterios de la OMS. Se constatan importantes avances en la aplicación del Convenio Marco de la OMS para el Control del Tabaco en las Américas. Al 2020, la mayoría de los países contaban con normativas sobre ambientes 100% libres de humo en lugares cerrados públicos y de trabajo, y el transporte público, y advertencias sanitarias gráficas grandes en los paquetes de tabaco. Desde el 2016 se duplicó el número de países que prohíben la publicidad, la promoción y el patrocinio del tabaco y que aplican impuestos al tabaco al nivel mínimo recomendado por la OMS. Sin embargo, aún no se ha alcanzado la meta prevista al 2022 para ninguna de esas medidas ni para la ratificación de los tratados internacionales en el tema. Aunque se ha avanzado en la Región, el avance no ha sido uniforme, y a menos que el ritmo de aplicación de las medidas de control del tabaco contenidas en la Estrategia y Plan de Acción se acelere, es poco probable que se logren las metas establecidas. La interferencia de la industria tabacalera se mantiene como uno de los principales retos.

## Abstract (en)

This report describes the current status of the tobacco control measures contained in the Strategy and Plan of Action to Strengthen Tobacco Control in the Region of the Americas 2018-2022 (Pan American Health Organization) and the advances made in its application, identifying achievements from 2016 to 2020 and challenges that still need to be addressed in order to reach the expected goals. This analysis relied on data from the World Health Organization (WHO) Report on the Global Tobacco Epidemic from 2015, 2017, and 2019, and national regulations were analyzed to determine their consistency with WHO criteria. Significant progress has been made in implementation of the WHO Framework Convention on Tobacco Control in the Americas. By 2020, most countries had regulations on 100% smoke-free environments in indoor public places, workplaces, and public transport, and large graphic health warnings on tobacco packaging. The number of countries that ban tobacco advertising, promotion, and sponsorship and that tax tobacco at the minimum level recommended by WHO has doubled since 2016. However, the 2022 targets have not yet been reached for any of these measures or for ratification of the relevant international agreements. Although progress has been made in the Region, it has not been uniform. Unless the pace of application of the tobacco control measures contained in the Strategy and Plan of Action accelerates, it is unlikely that its targets will be met. Tobacco industry interference remains one of the main challenges.

## RESUMO

Este artigo descreve a situação atual e o progresso na implementação das medidas para o controle do tabagismo prescritas na Estratégia e plano de ação para fortalecer o controle do tabagismo na Região das Américas 2018-2022, reconhece as conquistas realizadas no período entre 2016 e 2020, e identifica os desafios a serem enfrentados para alcançar as metas planejadas. A análise se baseou em dados obtidos do Relatório da OMS sobre a Epidemia Global do Tabaco, publicado em 2015, 2017 e 2019, e em regulamentações nacionais para determinar o cumprimento dos critérios da OMS. Observam-se avanços na implementação da Convenção-Quadro da OMS para o Controle do Tabaco nas Américas. Em 2020, a maior parte dos países dispunha de regulamentações para ambientes 100% livres da fumaça do tabaco em locais públicos fechados, locais fechados de trabalho e meios de transporte público, e advertências sanitárias com ilustrações gráficas grandes nas embalagens dos produtos de tabaco. O número de países que proíbem publicidade, promoção e patrocínio do tabaco e adotaram impostos sobre os produtos do tabaco no padrão mínimo recomendado pela OMS dobrou desde 2016. No entanto, as metas planejadas para 2022 ainda não foram atingidas – tanto em relação a estas medidas quanto à ratificação dos respectivos tratados internacionais. Apesar dos avanços, o progresso na Região não é uniforme. Se as medidas para o controle do tabaco prescritas na Estratégia e plano de ação não forem implementadas em um ritmo acelerado, as metas dificilmente serão alcançadas. A interferência da indústria do tabaco continua sendo um dos maiores desafios.

El consumo de tabaco es responsable de casi 1 millón de muertes cada año en la Región de las Américas (1) y constituye el único factor de riesgo común de las cuatro principales enfermedades no transmisibles (ENT): enfermedades cardiovasculares, cáncer, enfermedades respiratorias crónicas y diabetes. Además, impone una considerable carga a las economías nacionales (2) y los hogares —particularmente a los más pobres y vulnerables (3)—, y al medio ambiente (4). Tomando en cuenta la amenaza que la epidemia de tabaquismo representa para el desarrollo sostenible, así como la contribución que las medidas de control del tabaco pueden ejercer para minimizarla, entre las metas del Objetivo 3 de la Agenda 2030 de las Naciones Unidas para el Desarrollo Sostenible se establece fortalecer la aplicación del Convenio Marco de la Organización Mundial de la Salud para el Control del Tabaco (CMCT) en todos los países (meta 3a), con el fin de reducir la mortalidad prematura debida a ENT en un tercio para el 2030 (meta 3.4) (5).

Las medidas efectivas para combatir la epidemia de tabaquismo están contenidas en el CMCT, que es el primer tratado internacional de salud pública negociado bajo los auspicios de la Organización Mundial de la Salud (OMS) (6). Desde su entrada en vigor en el año 2005, el CMCT ha dinamizado la acción en este tema en las Américas, aunque la aplicación de las medidas contenidas en él no ha sido uniforme. En el 2017, se constató una tendencia al enlentecimiento en la aplicación de las medidas contenidas en el CMCT (7), lo que motivó que los 35 Estados Miembros de la Organización Panamericana de la Salud (OPS) aprobaran unánimemente la Estrategia y Plan de Acción para Fortalecer el Control del Tabaco en la Región de las Américas 2018-2022 (en lo adelante Estrategia y Plan de Acción) durante la 29.^a^ Conferencia Sanitaria Panamericana (8).

La Estrategia y Plan de Acción está alineada con el Plan de Acción Mundial para la Prevención y el Control de las Enfermedades No Transmisibles 2013-2020 (9) y el Plan de Acción para la Prevención y el Control de las Enfermedades No Transmisibles en las Américas 2013-2019 (10), así como con la Agenda 2030 de las Naciones Unidas para el Desarrollo Sostenible (5). Su objetivo general es acelerar la aplicación del CMCT en la Región, especialmente de los artículos referidos a las medidas que la OMS considera como “mejores inversiones”, es decir, las más eficaces en relación con su costo y que resultan más factibles de aplicar para prevenir las ENT (11).

Dada la magnitud de la epidemia de tabaquismo y sus implicaciones sanitarias, sociales y económicas, es necesario monitorear la aplicación de las medidas de control del tabaco en la Región de las Américas. En este estudio se describen el estado actual y los avances en la aplicación de las medidas de control del tabaco contenidas en la Estrategia y Plan de Acción —y otros compromisos globales relacionados— en los ámbitos nacionales y se identifican los logros alcanzados entre los años 2016 y 2020 y los retos que aún se deben enfrentar para cumplir las metas previstas.

## MATERIALES Y MÉTODOS

El análisis se centró en los avances logrados en las cuatro líneas estratégicas de acción y los siete objetivos contenidos en la Estrategia y Plan de Acción (cuadro 1), tomando como línea base el estado de la aplicación de las medidas correspondientes a cada línea estratégica de acción en el 2016 y sus 10 indicadores. A partir de esa línea de base, se describió el progreso alcanzado hasta el 30 de junio del 2020 para lograr las metas establecidas para el 31 de diciembre del 2022, según lo acordado por todos los Estados Miembros de la OPS (8).

**CUADRO 1.** Líneas estratégicas de acción y objetivos de la Estrategia y Plan de Acción para Fortalecer el Control del Tabaco en la Región de las Américas 2018-2022

Línea estratégica de acción	Objetivo
1. Aplicar medidas para el establecimiento de ambientes completamente libres de humo de tabaco (artículo 8 del CMCT) y para la adopción de medidas efectivas sobre el empaquetado y etiquetado de los productos de tabaco (artículo 11 del CMCT) como una prioridad para la Región	1.1. Adoptar legislación de ambientes libres de humo de tabaco en toda la Región de las Américas (medida P del MPOWER) 1.2. Incluir advertencias sanitarias en el empaquetado de los productos de tabaco (medida W del MPOWER)
2. Implementar la prohibición de la publicidad, la promoción y el patrocinio de tabaco (artículo 13 del CMCT) y adoptar medidas para reducir la asequibilidad a productos de tabaco (artículo 6 del CMCT)	2.1. Adoptar la prohibición total de la publicidad, la promoción y el patrocinio del tabaco (medida E del MPOWER) 2.2. Reducir la asequibilidad de los productos de tabaco mediante el aumento de los impuestos al consumo de tabaco (medida R del MPOWER)
3. Ratificar el CMCT y el Protocolo para la Eliminación del Comercio Ilícito de Productos de Tabaco por parte de los Estados Miembros de la OPS que aún no lo han hecho	3.1. Lograr la ratificación del CMCT 3.2. Lograr la ratificación del Protocolo (artículo 15 del CMCT)
4. Fortalecer la capacidad de los Estados Miembros de la OPS en las políticas de salud pública para hacer frente a los intentos de interferencia de la industria tabacalera y de los que defienden sus intereses	4.1. Establecer mecanismos eficaces para evitar la interferencia de la industria tabacalera y de los que defienden sus intereses (artículo 5.3 del CMCT)

***Fuente:*** Preparado por los autores a partir de la Estrategia y Plan de Acción para Fortalecer el Control del Tabaco en la Región de las Américas 2018-2022 (8).

***Nota:*** CMCT: Convenio Marco de la Organización Mundial de la Salud para el Control del Tabaco; OPS: Organización Panamericana de la Salud; MPOWER: plan de medidas para hacer retroceder la epidemia de tabaquismo (12), descritas en el cuadro 2.

En el análisis se incluyeron también los avances logrados desde el 2016 en la aplicación de las dos medidas del paquete de medidas efectivas para prevenir y reducir el consumo de tabaco MPOWER (12) que no se incluyeron en las cuatro líneas estratégicas de acción de la Estrategia y Plan de Acción, descritas en el cuadro 2: la medida M, relacionada con el monitoreo del consumo de tabaco y de las políticas de prevención, y la medida O, sobre la oferta de ayuda para el abandono del tabaco. Si bien estas dos medidas no están entre las consideradas por la OMS como “mejores inversiones”, reflejan dos aristas de gran importancia que se deben tomar en cuenta.

### Fuente de datos

Para este estudio se utilizaron los datos del Informe de la OMS sobre la Epidemia Mundial de Tabaquismo (en lo adelante Informe Mundial) del 2019 (13), sus versiones anteriores correspondientes al 2015 (14) y al 2017 (15), las normativas nacionales recolectadas por el equipo de control del tabaco del departamento de Enfermedades No Transmisibles y Salud Mental, de la OPS, (en lo adelante equipo de control del tabaco de la OPS) y la base de tratados de las Naciones Unidas (16).

En el Informe Mundial, publicado con frecuencia bienal, se recogen las normativas nacionales —leyes, decretos, resoluciones, etc.— e informaciones de carácter oficial —como políticas y programas— referidas a las líneas estratégicas de acción 1 y 2 de la Estrategia y Plan de Acción. La recopilación de esta información se realiza mediante cuestionarios estructurados enviados a los Estados Miembros de la OMS a través de las oficinas regionales y de país. Adicionalmente, el equipo de control del tabaco de la OPS, mediante su labor de asistencia técnica, hace un seguimiento regular de la aprobación de nuevas normativas referidas a este tema y de los cambios en las ya existentes.

La fuente de información para las normativas referidas a la línea estratégica de acción 3, fue la base de tratados de las Naciones Unidas (16). Al momento del análisis, no se contaba con información disponible para la línea estratégica de acción 4. La recopilación de estos datos se está finalizando en el 2021 en el marco del proceso de elaboración del Informe Mundial 2021 (17).

**CUADRO 2.** Correspondencia de las medidas del paquete MPOWER con los artículos del CMCT, las medidas consideradas “mejores inversiones” por la OMS y las líneas estratégicas de acción de la Estrategia y Plan de Acción para Fortalecer el Control del Tabaco en la Región de las Américas 2018-2022

Medidas del paquete MPOWER	Artículo del CMCT	Mejores inversiones, según la OMS	Línea estratégica de acción en la Estrategia y Plan de Acción 2018-2022
**M**(onitor): Monitorear el consumo de tabaco y las políticas de prevención	20	-	-
**P**(rotect): Proteger a la población del humo del tabaco	8	Sí	1
**O**(ffer): Ofrecer ayuda para abandonar el consumo de tabaco	14	-	-
**W**(arn): Advertir sobre los peligros del tabaco	11	Sí	1
**E**(nforce): Hacer cumplir las prohibiciones de publicidad, promoción y patrocinio del tabaco	13	Sí	2
**R**(aise): Aumentar los impuestos al tabaco	6	Sí	2

***Fuente:*** Preparado por los autores a partir de la Estrategia y Plan de Acción para Fortalecer el Control del Tabaco en la Región de las Américas 2018-2022 (8) y el Plan de Acción Mundial para la Prevención y el Control de las Enfermedades No Transmisibles 2013-2020 (9).

***Nota:*** MPOWER: plan de medidas para hacer retroceder la epidemia de tabaquismo (12); CMCT: Convenio Marco de la Organización Mundial de la Salud para el Control del Tabaco; OMS: Organización Mundial de la Salud.

### Procedimientos para el análisis de datos

Los criterios para evaluar si las medidas adoptadas siguen los criterios de la OMS están establecidos en las notas técnicas del Informe Mundial del 2019 (13). Para la presente evaluación se revisaron las normativas nacionales por parte de al menos dos expertos en control del tabaco; en el caso de las Américas, un experto era de la sede central de la OMS y otro del equipo de control del tabaco de la OPS. En relación con las encuestas de vigilancia y monitoreo del consumo de tabaco, se tomaron en cuenta solamente las de representatividad nacional.

En el caso de las normativas de ambientes 100% libres de humo, además de las normativas nacionales, se consideraron las normativas subnacionales correspondientes al primer nivel de gobierno por debajo del nacional en los casos de países federativos cuando en conjunto cubrían al menos el 90% de la población nacional.

## RESULTADOS DE LA EVALUACIÓN

**FIGURA 1.**
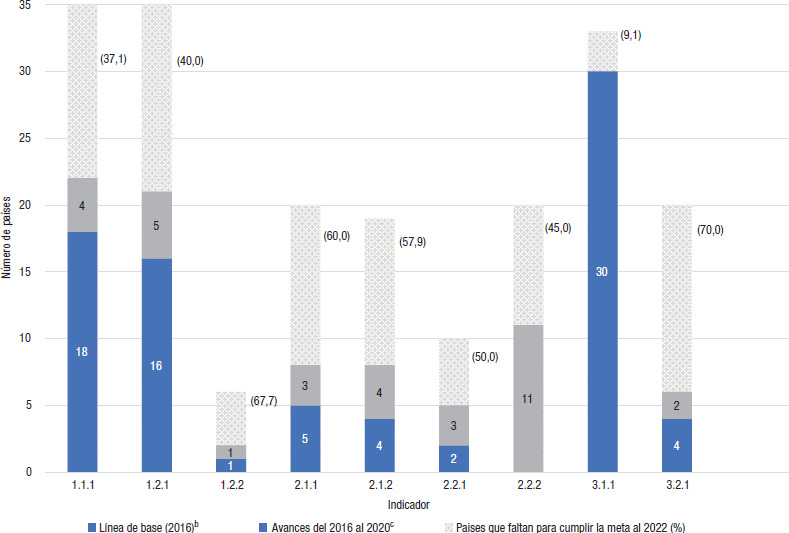
Resumen del avance de las principales medidas de control del tabaco en la Región de las Américas, 2016-2020^a^

Los resultados se presentan según los avances hacia el logro de las metas establecidas para cada una de las líneas estratégicas de acción en la Estrategia y Plan de Acción (cuadro 3; ver la figura 1 para visualizar mejor los resultados y comparar más fácilmente los indicadores entre sí) y en la aplicación de las medidas M y O del paquete MPOWER (cuadro 4).

### Línea estratégica de acción 1

La meta para el indicador 1.1.1 es que para el 2022 los 35 Estados Miembros de la OPS establezcan ambientes 100% libres de humo de tabaco en lugares públicos y de trabajo cerrados, y en el transporte público. Al 30 de junio del 2020, ya 22 países (que abarcan aproximadamente el 50% de la población de la Región) alcanzaron la meta, luego de sumarse cuatro países después del 2016.

Para el indicador 1.2.1, la meta al 2022 es que los 35 Estados Miembros de la OPS establezcan advertencias sanitarias que cubran al menos el 50% de las superficies principales de los paquetes de cigarrillos y cumplan con las características apropiadas (19). Al 30 de junio del 2020, 21 países (56% de la población) alcanzaron la meta, con la adhesión de cinco países desde el 2016.

Con respecto al indicador 1.2.2, la meta al 2022 es que seis países adopten ya sea la presentación única por marca (solo se permite la venta de una sola variante por marca) o el empaquetado neutro de productos de tabaco (prohibición del uso en el empaquetado de logotipos, colores, imágenes de marca o información promocional que no sea el nombre comercial o el nombre del producto en un color y tipo de letra corrientes) (20). Al 30 de junio del 2020, dos países (6,5% de la población) ya aplicaban el empaquetado neutro y uno de ellos establecía, además, la presentación única desde el 2016.

### Línea estratégica de acción 2

La meta para el indicador 2.1.1 para el 2022 es que 20 países apliquen la prohibición total de la publicidad, la promoción y el patrocinio del tabaco. Al 30 de junio del 2020, ocho países (30% de la población) habían alcanzado la meta, con la adición de tres países desde el 2016.

Para el indicador 2.1.2, la meta para el 2022 es que 19 países prohíban la exhibición de productos de tabaco en puntos de venta (solo se permite la enumeración textual de productos y sus respectivos precios, sin elementos promocionales) (19). Ocho países (9% de la población) habían alcanzado la meta al 30 de junio del 2020, con cuatro países incorporados desde el 2016.

Con relación al indicador 2.2.1, la meta para el 2022 es que 10 países logren que la carga tributaria del total de impuestos indirectos represente el 75% o más del precio de venta minorista de la marca más vendida de cigarrillos en el país o que aumente significativamente para promover un cambio en la categoría de carga tributaria total sobre cigarrillos, según los umbrales establecidos para la medida en el Informe Mundial del 2019 (13). De acuerdo con los últimos datos disponibles, al 31 de julio del 2018 cinco países (32% de la población) habían alcanzado la meta, con la adición de tres países desde el 2016.

Para el 2022, la meta del indicador 2.2.2 es que 20 países aumenten sus impuestos selectivos de forma tal que disminuya relativamente en 10% o más la asequibilidad de los cigarrillos en comparación con su asequibilidad en el 2014, según el índice de asequibilidad presentado en el Informe Mundial del 2015. Este índice se define como el porcentaje del producto interno bruto per cápita que se necesita para comprar 100 paquetes de la marca más vendida; mientras mayor es el valor del índice, menos asequibles son los cigarrillos (13). De acuerdo con los datos disponibles, al 31 de julio del 2018 ya 11 países (22,5% de la población) habían alcanzado la meta.

**CUADRO 3.** Resumen de los avances en la aplicación de la Estrategia y Plan de Acción para Fortalecer el Control del Tabaco en la Región de las Américas 2018-2022

Indicador	Línea de base, diciembre del 2016^a^	Avances entre diciembre del 2016 y junio del 2020^b^	Estado actual, junio del 2020^b^		Meta a diciembre del 2022
			Número de países	Población abarcada^c^ (%)	
**Línea estratégica de acción 1**					
1.1.1. Número de países con normativa nacional que establece ambientes 100% libres de humo de tabaco en todo lugar público y de trabajo cerrado, y en el transporte público	18 (Argentina, Barbados, Brasil, Canadá, Chile, Colombia, Costa Rica, Ecuador, El Salvador, Guatemala, Honduras, Jamaica, Panamá, Perú, Surinam, Trinidad y Tabago, Uruguay y Venezuela)	4 (Antigua y Barbuda, Bolivia, Guyana y Santa Lucía)	22	50,0	35
1.2.1. Número de países con advertencias sanitarias gráficas en el empaquetado del tabaco que cumplen con los criterios del Informe Mundial del 2019 (13)	16 (Argentina, Bolivia, Brasil, Canadá, Chile, Costa Rica, Ecuador, El Salvador, Jamaica, México, Panamá, Perú, Surinam, Trinidad y Tabago, Uruguay y Venezuela)	5 (Antigua y Barbuda^d^, Barbados, Guyana, Honduras y Santa Lucía)	21	56,0	35
1.2.2. Número de países que adoptan una política de empaquetado neutro y/o presentación única	1 (Uruguay, con presentación única)	1 (Canadá; Uruguay adoptó el empaquetado neutro)	2	6,5	6
**Línea estratégica de acción 2**					
2.1.1. Número de países con prohibición total de la publicidad, la promoción y el patrocinio del tabaco	5 (Brasil, Colombia, Panamá, Surinam y Uruguay)	3 (Antigua y Barbuda, Guyana y Venezuela)	8	30,0	20
2.1.2. Número de países que incluyen en su prohibición de publicidad, promoción y patrocinio la prohibición de exhibir el producto en los puntos de venta	4 (Panamá, Surinam, Trinidad y Tabago^e^, y Uruguay)	4 (Colombia, Costa Rica^e^, Guyana y Venezuela)	8	9,0	19
2.2.1. Número de países en los que los impuestos totales representan el 75% o más del precio final de venta menorista, o el aumento ha sido significativo para promover un cambio de categoría en la carga tributaria total sobre cigarrillos	2 (Argentina y Chile)	3 (Brasil, Colombia y Guyana^f^)	5	32,0	10
2.2.2. Número de países con aumento de sus impuestos al consumo de tabaco de forma tal que se promueve un incremento del índice de asequibilidad presentado en el Informe Mundial del 2015^g^	0	11 (Argentina, Canadá, Chile, Colombia, Ecuador, Jamaica, Paraguay, Perú, República Dominicana, Trinidad y Tabago, y Uruguay)	11	22,5	20
**Línea estratégica de acción 3**					
3.1.1. Número de Estados Parte en el CMCT	30 (Antigua y Barbuda, Bahamas, Barbados, Belice, Bolivia, Brasil, Canadá, Chile, Colombia, Costa Rica, Dominica, Ecuador, El Salvador, Granada, Guatemala, Guyana, Honduras, Jamaica, México, Nicaragua, Panamá, Paraguay, Perú, Saint Kitts y Nevis, Santa Lucía, San Vicente y las Granadinas, Surinam, Trinidad y Tabago, Uruguay y Venezuela)	0	30	60,0	33
3.2.1. Número de Estados Parte en el CMCT y el PCIPT	4 (Ecuador, Nicaragua, Panamá y Uruguay)	2 (Brasil y Costa Rica)	6	25,0	20
**Línea estratégica de acción 4**					
4.1.1. Número de países con mecanismos de identificación y manejo de conflictos de intereses para funcionarios y empleados públicos con competencia en las políticas de control del tabaco	ND	ND	ND	ND	20

***Fuente:*** Preparado por los autores a partir del Informe de la Organización Mundial de la Salud sobre la Epidemia Mundial de Tabaquismo del 2019 (13), sus versiones anteriores correspondientes al 2015 (14) y al 2017 (15) y de los datos del estudio.

***Nota:*** CMCT: Convenio Marco de la Organización Mundial de la Salud para el Control del Tabaco; PCIPT: Protocolo para la Eliminación del Comercio Ilícito de Productos del Tabaco; ND: no disponible.

a La línea de base corresponde a la situación el 31 de diciembre del 2016 para los indicadores 1.1.1, 1.2.1, 1.2.2, 2.1.1, 2.1.2, 3.1.1 y 3.1.2, y a la situación el 31 de julio del 2016 para el indicador 2.2.1.

b Los avances para los indicadores 2.2.1 y 2.2.2 corresponden a los últimos datos disponibles al 31 de julio del 2018.

c El cálculo de la población cubierta para cada indicador se elaboró a partir de los datos disponibles de la población total al 1 de julio del 2019, según las Naciones Unidas (18). Para el denominador se consideró la suma de la población de los 35 Estados Miembros de la Organización Panamericana de la Salud.

d Antigua y Barbuda se incluye, aunque las regulaciones están pendientes.

e Costa Rica, y Trinidad y Tabago prohíben la exhibición de productos de tabaco en puntos de venta, pero su prohibición de la publicidad, la promoción y el patrocinio es incompleta (13).

f Guyana subió de la primera categoría de carga tributaria total sobre cigarrillos (0-25%) a la segunda (25-50%).

g El indicador 2.2.2 se cumple si un país aumentó sus impuestos selectivos al consumo de forma tal que disminuyó relativamente en 10% o más la asequibilidad de los cigarrillos en comparación con su asequibilidad en el 2014, medida mediante el índice de asequibilidad del Informe Mundial (15).

***Fuente:*** Preparado por los autores a partir de la Estrategia y Plan de Acción para Fortalecer el Control del Tabaco en la Región de las Américas 2018-2022 (8), del Informe de la Organización Mundial de la Salud sobre la Epidemia Mundial de Tabaquismo del 2019 (13) y de los datos del estudio.

a No se dispone de la información sobre el indicador 4.1.1. b La línea de base corresponde a la situación el 31 de diciembre del 2016 para los indicadores 1.1.1, 1.2.1, 1.2.2, 2.1.1, 2.1.2, 3.1.1 y 3.1.2, y a la situación el 31 de julio del 2016 para el indicador 2.2.1. c Avances entre diciembre del 2016 y junio del 2020; los avances en los indicadores 2.2.1 y 2.2.2 según los últimos datos disponibles al 31 de julio del 2018.

### Línea estratégica de acción 3

La meta para el indicador 3.1.1 para el 2022, es que 33 países hayan ratificado el CMCT. Al 30 de junio del 2020 se habían sumado 30 países (60% de la población) sin ninguna adición desde el 2016.

Con respecto al indicador 3.2.1, la meta para el 2022 es que 20 Estados Parte del CMCT ratifiquen el Protocolo para la Eliminación del Comercio Ilícito de Productos de Tabaco (21). Al 30 de junio del 2020, seis países (25% de la población) habían ratificado ese Protocolo, con la adhesión de dos países desde el 2016.

### Línea estratégica de acción 4

La meta para el indicador 4.1.1 para el 2022 es que 20 países cuenten con mecanismos de identificación y manejo de conflictos de intereses para los funcionarios y empleados públicos con competencia en las políticas de control del tabaco (19). En junio del 2021, aún no se contaba con información sobre el comportamiento de este indicador.

### Medida M de MPOWER

De acuerdo con la información disponible al 31 de julio del 2018, un total de 11 de los 35 países de la Región (65% de la población) cumplían con todos los requisitos de mejores prácticas en el monitoreo del consumo de tabaco y las políticas de prevención, según los criterios de la OMS; desde el 2016 se sumó un país (13).

### Medida O de MPOWER

Respecto a la oferta de ayuda para abandonar el consumo de tabaco, la última información disponible corresponde a lo publicado en el Informe Mundial del 2019, según la cual seis países de las Américas (73% de la población) mantenían al más alto nivel la aplicación de mejores prácticas según los criterios de la OMS, un país menos que en el 2016 (13).

**CUADRO 4.** Resumen de los avances en el monitoreo del consumo de tabaco y las políticas de prevención y oferta de ayuda para el abandono del tabaco en la Región de las Américas, 2016-2018

Otras medidas para el control del tabaco	Indicador	Línea de base, diciembre del 2016^a^	Estado actual, diciembre del 2018^b^ y cobertura poblacional (%)^c^
Monitorear el consumo de tabaco y las políticas de prevención (medida M de MPOWER)	Número de países que cuentan con sistemas de vigilancia para el control del tabaco que proveen datos recientes sobre la prevalencia del consumo de tabaco, tanto en adultos como en jóvenes, representativos de toda la población nacional, y que se generan periódicamente (al menos cada 5 años)	10 Argentina, Barbados, Brasil, Canadá, Chile, Colombia, Costa Rica, Estados Unidos de América, Panamá y Uruguay	11 Bahamas, Brasil, Canadá, Chile, Costa Rica, Ecuador, Estados Unidos de América, Panamá, Perú, Surinam y Uruguay (65%)
Ofrecer ayuda para abandonar el consumo de tabaco (medida O de MPOWER)	Número de países que cuentan con una línea telefónica nacional de cesación, y disponibilidad de servicios de cesación (en centros de salud de atención primaria, hospitales, consultorios de profesionales de la salud, de la comunidad, u otros) y de terapia de sustitución nicotínica, y que al menos uno de estos dos servicios cuenta con cobertura de costos	7 Brasil, Canadá, El Salvador, Estados Unidos de América, Jamaica, Panamá^d^ y México	6 Brasil, Canadá, El Salvador, Estados Unidos de América, Jamaica y México (71%)

***Fuente:*** Preparado por los autores a partir del Informe de la OMS sobre la Epidemia Mundial de Tabaquismo del 2019 (13).

***Notas:*** MPOWER: plan de medidas para hacer retroceder la epidemia de tabaquismo (12), descritas en el cuadro 2.

a La situación en diciembre del 2016 para las medidas M y O del paquete MPOWER corresponde a los datos disponibles al 31 de diciembre del 2016.

b El avance para las medidas M y O del paquete MPOWER corresponde a los datos disponibles al 31 de diciembre del 2018.

c El cálculo de la cobertura poblacional por cada medida se estimó a partir de los datos de la población total al 1 de julio del 2019, según las Naciones Unidas (18). Para esta publicación se consideró como población total la suma de la población de los 35 Estados Miembros de la Organización Panamericana de la Salud.

d Del 2016 al 2018, la línea telefónica de ayuda para la cesación del uso de tabaco de Panamá dejo de operar.

## LOGROS ALCANZADOS EN LA REGIÓN

Desde la aprobación de la Estrategia y Plan de Acción, se constatan importantes avances en la aplicación del CMCT en las Américas. En particular, en los últimos años se ha observado un significativo progreso —aunque no homogéneo— en la subregión del Caribe no hispanohablante. En Antigua y Barbuda, Guyana y Surinam se aprobaron normativas de control del tabaco, como el establecimiento de ambientes libres de humo de tabaco, el apropiado empaquetado y etiquetado de los productos de tabaco, y la prohibición completa de la publicidad, la promoción y el patrocinio del tabaco, medidas consistentes con los artículos 8, 11 y 13 del CMCT, respectivamente. No obstante, los países del Caribe no hispanohablantes siguen rezagados en otros importantes aspectos, ya que en seis de esos 14 países no se ha adoptado aún ninguna medida del paquete MPOWER al nivel de mejores prácticas según los criterios de la OMS.

En los países sudamericanos también se advierten avances en el período analizado. En el 2018, Brasil se convirtió en el segundo país del mundo —después de Turquía— en aplicar las seis medidas del paquete MPOWER al nivel de mejores prácticas. Eso representa un logro importante, dado que las medidas de control del tabaco potencian su impacto positivo cuando se implementan en conjunto, tal como fue originalmente concebido en el CMCT. La implementación de las medidas al nivel de mejores prácticas que ha venido ocurriendo gradualmente en Brasil desde el 2002, se ha traducido en una disminución en la prevalencia de fumadores de cigarrillos en las capitales del país de 15,6% en el 2007 a 9,8% en el 2019 (22). Asimismo, con la adopción en Bolivia de la ley que establece ambientes 100% libres de humo de tabaco en todos los lugares públicos y de trabajo cerrados, y en el transporte público, y la aprobación por Paraguay de una enmienda a su ley de control del tabaco —que no está contabilizada en los resultados de este análisis por haber ocurrido después de la fecha de corte de la recopilación de la información—, la América del Sur se convirtió en 2020 en una subregión 100% libre de humo de tabaco. A pesar de ello, el cumplimiento de estas normativas continúa siendo un desafío, ya que —como se refleja en el Informe Mundial del 2019— una evaluación cualitativa de expertos nacionales mostró que el nivel promedio de cumplimento es moderado en 20 países (13).

En el caso de los países de América del Norte, un avance significativo ha sido la aprobación del empaquetado neutro de productos de tabaco en Canadá, país que se suma a Uruguay en el liderazgo en este tema.

Todos estos avances en la aplicación de medidas de control del tabaco en los países de la Región han contribuido a que, según estimaciones de la OMS —y a diferencia de la proyección a nivel mundial—, la Región de las Américas alcanzaría la meta propuesta en el Plan de Acción Mundial para la Prevención y el Control de las Enfermedades No Transmisibles 2013-2020 de lograr una disminución relativa de 30% en la prevalencia de consumo de tabaco en adultos para el 2025 (23). Asimismo, dado que el tabaco es el factor de riesgo de ENT más sensible a políticas específicas, los países de la Región podrían contribuir aun más significativamente a la reducción de la prevalencia y la mortalidad prematura por ENT en el mundo si lograran reducciones relativas por encima del 30% (24).

## RETOS Y PERSPECTIVAS

A pesar de los avances descritos más arriba, a menos que el ritmo de aplicación de las medidas de control del tabaco contenidas en la Estrategia y Plan de Acción se acelere, es poco probable que en la Región de las Américas se logren las metas establecidas para el 2022.

La medida referida a aplicar la prohibición total de la publicidad, la promoción y el patrocinio del tabaco se mantiene entre las medidas menos aplicadas en la Región. Además, a pesar de que los impuestos al tabaco representan la medida más efectiva en función del costo para reducir su consumo (25), sigue siendo ampliamente subutilizada en la Región. Esto se explica en gran parte por la interferencia de la industria tabacalera, que trata de impedir su aplicación arguyendo que aumentar los impuestos selectivos al consumo de tabaco lleva necesariamente al aumento del comercio ilícito de estos productos. En realidad, la evidencia muestra que los impuestos y precios tienen un efecto limitado en el comercio ilícito y que otros factores —como los relacionados con la gobernanza y el control de la cadena de suministro de los productos de tabaco— están entre los principales determinantes de ese fenómeno (26, 27). Asimismo, estudios robustos, transparentes e independientes de medición del tamaño del comercio ilícito de cigarrillos en la Región han encontrado que las cifras usadas por la industria tabacalera tienden a estar sobreestimadas (28-30). El Protocolo para la Eliminación del Comercio Ilícito de Productos de Tabaco es la mejor respuesta al comercio ilegal de esos productos (21) y su ratificación es un reto en sí mismo, pues solo seis países de la Región se han unido a él, con poco avance en los últimos años.

Otro de los escollos para avanzar en la aplicación del CMCT es la inexistencia en muchos países de medidas destinadas a neutralizar los intentos de la industria tabacalera de interferir, demorar, obstaculizar o impedir la aplicación de las medidas de control del tabaco orientadas a proteger la salud de la población. También se deben dedicar mayores y más sostenidos esfuerzos a monitorear, documentar y hacer públicas las actividades de la industria tabacalera que ponen en evidencia sus estrategias obstructivas, y a la adopción de instrumentos legales para abordar posibles conflictos de intereses por parte de los funcionarios involucrados en el control del tabaco.

Estrechamente vinculado a ello, está el desafío de redoblar esfuerzos hacia el establecimiento de mecanismos coordinadores nacionales dirigidos al control del tabaco —en consonancia con el artículo 5.2 del CMCT— que cuenten con el debido financiamiento y faciliten la coherencia entre las políticas sanitarias y las fiscales, comerciales, agrícolas, educativas y cualquier otra necesaria para dar una respuesta efectiva e integral a la epidemia de tabaquismo.

El fortalecimiento de la capacidad técnica en el ámbito nacional para desarrollar investigaciones y generar evidencias que permitan sustentar políticas nacionales y, principalmente, revertir los argumentos obstaculizadores de la industria tabacalera y sus aliados es otro reto transversal que enfrenta la Región (31). En este sentido, es importante continuar fomentando la cooperación Sur-Sur, así como el intercambio de conocimientos y experiencias entre los países en foros regionales y subregionales.

Estos desafíos se hacen aun más urgentes en el contexto de la pandemia de la COVID-19. Por un lado, hay información de que la industria tabacalera ha aprovechado la pandemia para redoblar la promoción de sus productos e intentar posicionarse como un interlocutor válido en la discusión de políticas públicas (32); mientras por otro lado, la evidencia disponible señala que las personas con ENT y los fumadores tienen mayor riesgo de enfermar gravemente y de morir si contraen la COVID-19 (33, 34).

En el contexto de esta pandemia se hace impostergable la adopción de políticas contundentes para enfrentar los intentos de interferencia de la industria tabacalera y es imprescindible contar con una fluida interacción entre los distintos sectores de gobierno involucrados en los diversos ámbitos del control del tabaco, mediante la pronta implementación de los mecanismos de coordinación nacional mencionados. La difícil realidad sanitaria impuesta por la COVID-19, en la que los países podrían necesitar recursos adicionales para responder a la pandemia, podría ayudar a posicionar el papel de los impuestos a productos malsanos —como los productos de tabaco— en los planes de recuperación económica, ya que constituyen una fuente de recaudación tributaria adicional e inmediata. Asimismo, esta situación puede abrir una oportunidad para fortalecer los servicios de ayuda para el abandono del tabaco en el primer nivel de atención, en el marco de una transformación en el abordaje de las ENT y sus factores de riesgo a partir de la pandemia. Finalmente, se podrían fortalecer los sistemas de vigilancia de la prevalencia para investigar, entre otros temas de importancia, cómo la pandemia ha impactado en la epidemia de tabaquismo.

## CONCLUSIONES

La aplicación del CMCT sigue avanzando en la Región de las Américas y se han registrado importantes avances en el Caribe no hispanohablante, América del Sur y América del Norte en los últimos años; sin embargo, los logros han sido muy escasos en los países de Centroamérica y el Caribe hispanohablante. Se debe hacer notar, no obstante, que a pesar de lo alcanzado en el Caribe no hispanohablante, esta sigue siendo la subregión donde el nivel de aplicación de medidas efectivas es menor que la esperada.

En el 2020, la mayoría de los países contaban con ambientes 100% libres de humo de tabaco en lugares cerrados públicos y de trabajo, y el transporte público, además de advertencias sanitarias gráficas grandes en los paquetes de tabaco. Sin embargo, la Región se encuentra lejos de la meta trazada con vistas al 2022, a pesar de que gran parte de estas medidas son competencia exclusiva del sector de la salud.

Si bien aumentó el número de países en los que se aplica la prohibición de la publicidad, la promoción y el patrocinio, y una carga tributaria sobre productos de tabaco al nivel mínimo recomendado por la OMS, la realidad es que aún se está lejos de llegar a la meta esperada en el 2022. Igualmente, hubo un cambio mínimo en el número de países que monitorean el consumo de tabaco y un ligero retroceso en la oferta de ayuda para el abandono de su uso. Después del 2016, ningún otro país ha ratificado el CMCT y solamente dos ratificaron el Protocolo para la Eliminación del Comercio Ilícito de Productos de Tabaco.

Si se mantiene el ritmo actual de avance en la aplicación de medidas de control del tabaco, es poco probable que se logren las metas establecidas en la Estrategia y Plan de Acción para el 2022. La interferencia de la industria tabacalera se mantiene como uno de los principales obstáculos a vencer, en particular durante la pandemia de COVID-19 que hace más vulnerables a las personas con ENT y los fumadores. La situación actual de pandemia crea una oportunidad y una obligación para redoblar los esfuerzos en el combate contra la epidemia de tabaquismo y para contribuir a alcanzar las metas de la Agenda 2030 para el Desarrollo Sostenible en la Región de las Américas.

## Declaración.

Las opiniones expresadas en este artículo son responsabilidad de los autores y no necesariamente reflejan las opiniones, políticas o posiciones oficiales de las instituciones a las cuales están afiliadas, ni los criterios ni la política de la *Revista Panamericana de Salud Pública / Pan American Journal of Public Health* y/o de la Organización Panamericana de la Salud.
